# Use of Haploid Model of *Candida albicans* to Uncover Mechanism of Action of a Novel Antifungal Agent

**DOI:** 10.3389/fcimb.2018.00164

**Published:** 2018-06-08

**Authors:** Thuyen Truong, Tanujaa Suriyanarayanan, Guisheng Zeng, Thuc D. Le, Lin Liu, Jiuyong Li, Cao Tong, Yue Wang, Chaminda J. Seneviratne

**Affiliations:** ^1^Oral Sciences, Faculty of Dentistry, National University of Singapore, Singapore, Singapore; ^2^Institute of Molecular and Cell Biology, Agency for Science, Technology and Research, Singapore, Singapore; ^3^School of Information Technology and Mathematical Sciences, University of South Australia, Adelaide, SA, Australia; ^4^Department of Biochemistry, Yong Loo Lin School of Medicine, National University of Singapore, Singapore, Singapore

**Keywords:** *Candida albicans*, haploid, SM21, mechanism of action, mitochondria

## Abstract

Antifungal agents for the treatment of *Candida albicans* infections are limited. We recently discovered a novel antifungal small molecule, SM21, with promising *in vivo* activity. Herein, we employed the newly developed *C. albicans* haploid toolbox to uncover the mechanism of action of SM21. Comprehensive RNA-Seq analyses of the haploid susceptible GZY803 strain revealed significant gene expression changes related to mitochondria when exposed to SM21. Mitochondrial structure visualization and measurement of ATP generation, reactive oxygen species (ROS) levels, and the antioxidant potential of SM21-treated and untreated GZY803, mitochondrial structure defective haploid mutant (*dnm1*Δ), and wild-type diploid SC5314 strains confirmed defects in mitochondria. Exploiting the advantage of *C. albicans* haploids as a single ploidy model, we further exposed GZY803 to repetitive treatments of SM21 in order to generate resistant mutants. Three colonies designated S3, S5 and S6, which displayed resistance to SM21, were isolated. All resistant strains exhibited enhanced transcriptomic responses for peptide and protein metabolism and secreted aspartate proteases (SAPs) activity under SM21 treatment compared to the parent strain GZY803. Consistently, supplementing the resistant strains, GZY803, and SC5314 with peptone, a form of digested peptides, decreased susceptibility to SM21. The present study demonstrates the usefulness of haploid *C. albicans* model in antifungal drug discovery. The findings will be invaluable to develop SM21 as a novel antifungal agent, which will benefit millions of patients suffering from *Candida* infections.

## Introduction

*Candida albicans* is the most prevalent fungal pathogen in humans and ranked as the fourth leading cause of nosocomial blood-stream infections (Ashman et al., 2004). Systemic candidiasis is reported to have a mortality rate as high as 40% (Wisplinghoff et al., 2004). Moreover, *C. albicans* also causes superficial mucosal infections such as oral and vaginal candidiasis which are highly prevalent and persistent (Webb et al., 1998; Williams and Lewis, 2011). However, currently available antifungals for clinical uses are limited. Hence, there has been an urgent need to find novel anti-*Candida* agents for clinical uses. Our group recently discovered a new antifungal small molecule, designated SM21, which exhibited promising activity *in vitro* as well as *in vivo* (Wong et al., 2014). However, the mechanism by which SM21 exerts its antifungal activity remained elusive.

Until recently, *C. albicans* was thought to be an obligate diploid organism (Jones et al., 2004; Noble and Johnson, 2007). The invariably diploid and heterozygous nature of the organism was a considerable barrier for genetic studies in identifying novel gene functions and novel drug targets (Hickman et al., 2013). In addition, diploid model of *C. albicans* also poses a challenge for developing resistant mutations, which is a common approach to studying antifungal resistance of the organism (Woodford and Ellington, 2007). Generating resistant mutations in diploid *C. albicans* is difficult, as simultaneous inactivation of both copies of a gene is often required to produce a resistant phenotype, which is an extremely rare event. For instance, it takes about 100 days on average to generate a mutation resistant to echinocandin under optimized environmental conditions (Shields et al., 2015).

Existence of haploid forms of *C. albicans* was discovered in 2013 by Hickman et al. (2013). Single genome of haploid *C. albicans* could provide numerous advantages in biological and drug discovery research. Subsequently, our group demonstrated the utility of haploid model of *C. albicans* in uncovering novel molecular regulators in *C. albicans* biofilm formation and antifungal drug resistance (Seneviratne et al., 2015; Truong et al., 2016). Some haploid *C. albicans* strains were found to be able to maintain the haploidy in planktonic as well as biofilm cultures (Hickman et al., 2013; Seneviratne et al., 2015). In addition, we also demonstrated that *C. albicans* haploids contain a highly similar proteome to that of the diploids (Truong et al., 2016). Haploid cells have been shown to be more efficient in acquiring beneficial mutations at high doses of drug treatment when compared to that of the diploids in the yeast *Saccharomyces cerevisiae* (Anderson et al., 2004). Hence, we hypothesized that *C. albicans* haploid model could be a very useful tool to uncover drug targets of novel antifungal agents and resistance mechanisms.

Taking foregoing advantages into account, in the present study, we utilized haploid model of *C. albicans* to study the mechanism of action of the antifungal molecule SM21 and the resistance strategies of *C. albicans* against it. Firstly, transcriptomic analysis of the haploid *C. albicans* parent strain GZY803 in response to SM21 revealed diminished gene expression in mitochondrial ATP-coupled electron transport, respiration, and enhanced cytochrome assembly factors. Downstream analysis confirmed that SM21 treatment results in mitochondrial damage and inhibition of mitochondrial activity. Secondly, we generated *C. albicans* SM21-resistant haploid strains and performed transcriptomic analysis on the cultures with or without SM21 treatments. RNA-Seq analysis coupled with downstream experiments suggested the association between amino acid supplement and the resistance responses of *C. albicans* against SM21. Hence, the present study demonstrates a new application of the *C. albicans* haploid model in examining the drug targets of a novel antifungal and predicting possible resistance strategy by which the fungal pathogen may develop against it. The findings will aid future development of SM21 as a novel antifungal agent against *C. albicans* infections.

## Materials and methods

### Strains, plasmids, and culture conditions

Generation of *C. albicans* haploid strain (GZY803) was described previously (Hickman et al., 2013). Construction of other yeast strains and plasmids used in this study are described in Tables S3, S4, respectively. Unless specified, *C. albicans* cells were grown at 30°C in YPD (2% yeast extract, 1% peptone, and 2% glucose), or GMM (glucose minimal medium, 6.79 g/l yeast nitrogen base without amino acids, and 2% glucose) supplemented with appropriate amino acids (uridine 80 μg/ml, arginine 40 μg/ml, and histidine 40 μg/ml) and 5-FOA (1 mg/ml) when required. Solid culture plates were obtained by adding 2% agar into the media.

### Antifungal agents

SM21 (ChemBridge) Caspofungin, Amphotericin B, Fluconazole, Ketoconazole, and Voriconazole (Sigma) were used in this study.

### Antifungal susceptibility testing for *C. albicans* cells

Antifungal susceptibility tests of *C. albicans* cells was performed as described previously (Truong et al., 2016) following the Clinical and Laboratory Standards Institute's (CLSI) guideline. In brief, *C. albicans* cell suspension of 10^7^ cells/ml (equivalent to 0.38 McFarLand standards) was first prepared from overnight culture at 30°C. Subsequently, the culture was diluted with RPMI to yield an inoculum of ~10^3^ cells/ml. The MIC was determined in 96-well plates and each strain was exposed to the 2-fold diluted solutions of SM21. The plates were incubated at 30–35°C for 48 h before MIC values were determined following the CLSI guideline.

### Generation of SM21-resistant haploid strain

To induce spontaneous resistance to SM21 in haploid *C. albicans* strains, an inoculum of 10^8^ cells/ml of GZY803 was cultured in 2 μg/ml of SM21 in GMM for 3 consecutive days in 30°C. Cultures were replenished with fresh SM21 in GMM every 24 h. Subsequently, cells were pelleted and spread on GMM plates containing 4 μg/ml of SM21 and incubated at 30°C until colonies developed. All colonies developed were selected and examined for their MIC90 to SM21. Three colonies, labeled as S3, S5, and S6, which display an SM21 MIC of 4 μg/ml, were selected for further studies.

### RNA extraction and sequencing

RNA was extracted from GZY803 and the SM21-resistant strains, S3, S5, and S6, untreated or treated with 2 μg/ml of SM21 in GMM supplemented with required amino acids for 1 or 4 h at 30°C. Experiments were performed in three biological replicates for each strain in each condition. The RNA extraction was conducted following Ribopure RNA purification protocol (Qiagen); and samples were sent to Novogene company for quality check and RNA sequencing using Illumina HiSeq 4000 platform. Preliminary analysis was performed by Novogene.

### Ploidy analysis by flow cytometry

Ploidy of *C. albicans* strains was analyzed by flow cytometry as described (Zeng et al., 2014). In brief, *C. albicans* cells from 30°C cultures were washed with PBS, and fixed with 70% ethanol at 4°C overnight. Cells were then treated with RNase A (5 mg/ml) at 37°C for 6–7 h or overnight, washed with PBS, and further incubated with propidium iodine (5 mg/ml) in dark at 4°C overnight. Subsequently, stained samples were sonicated once to separate clustered cells, and subjected to DNA content analysis using BD FACSCalibur (BD Biosciences). Data acquisition was performed with 10,000 cells for each sample by using the CellQuest Pro software. The acquired data were then analyzed using WinMDI (version 2.8) software. Diploid (SC5314) and haploid (GZY803) cells were used as the control.

### ATP measurement

ATP generation was measured using BacTiter-Glo™ Microbial Cell Viability Assay (Promega, Singapore). Briefly, 10^7^ cells of mid-log phrase cultures after treatment with 2 μg/ml of SM21 for 4 h were measured for ATP generation. Untreated cells were included as a control. An aliquot of treated and untreated cells were subjected to colony forming unit (CFU) assay as described before (Truong et al., 2016) to normalize for total cells count in the cultures.

### Total antioxidant potential measurement

Total antioxidant potential estimation was described previously (Truong et al., 2016). *C. albicans* cultures at mid-log phrase (10^7^ cells/ml) were homogenized using glass beads (0.5 mm) and a high speed vortex (Vortexgene) following 3 cycles of 3 min on and 2 min off ice. Total antioxidant capacity assay kit (Abcam) was used to estimate the antioxidant potentials of the cultures following manufacture protocol.

### Florescent microscopy analysis

Protocol for visualization of mitochondrial structures was adapted from previous publication (Mavrianos et al., 2013). Briefly, an inoculum of 10^7^ *C. albicans* cells per ml were treated with 25 nM of MitoTracker Red CMXRos or MitoSOX™ Red Mitochondrial Superoxide Indicator (Invitrogen) for 20 min and the cells were visualized under an Olympus Fluoview FV1000 TIRF confocal microscope. Images were analyzed using Olympus FV10-ASW Viewer software.

### Differential expression analysis

For each strain, genes which showed a positive log2-fold change in both 1 h and 4 h SM21 treated conditions and with an average of larger than or equal to 1 in the two conditions as compared to the non-treated control were labeled as “higher.” Meanwhile, genes with negative log2-fold changes with an average fold change less than or equal to −1 were classified as “lower.” On the other hand, to identify genes which were commonly and similarly regulated in the resistant conditions but were differently expressed in the susceptible strain, we applied differential expression Limma method (Bioconductor) (Ritchie et al., 2015). Among the significantly altered genes with a *p* < 0.05, those which showed an average log2 fold change of larger than or equal to 1 were classified as “higher;” while those with an log2 average fold change of less than or equal to −1, “lower,” in the resistant strains as compared to the wild type.

### Cluster display and gene ontology analysis

Cluster display of *C. albicans*' gene expression was generated using Cluster (version 3.0) and TreeView (version 1.1.6) software (Eisen et al., 1998). Biological processes and cellular pathways which were significantly altered in the samples were illustrated using Cytoscape (version 2.8.3) (Shannon et al., 2003) with BINGO plugin (version 2.44) (Maere et al., 2005).

### Statistical analysis

Statistical analysis was performed using Student *t*-test. Analysis was done using SPSS software (Version 16.0, SPSS Inc.). The level of significance was set at *p* < 0.05.

## Results

### Transcriptomics analysis of haploid *C. albicans* exposed to SM21 treatment

**Figure 1:**
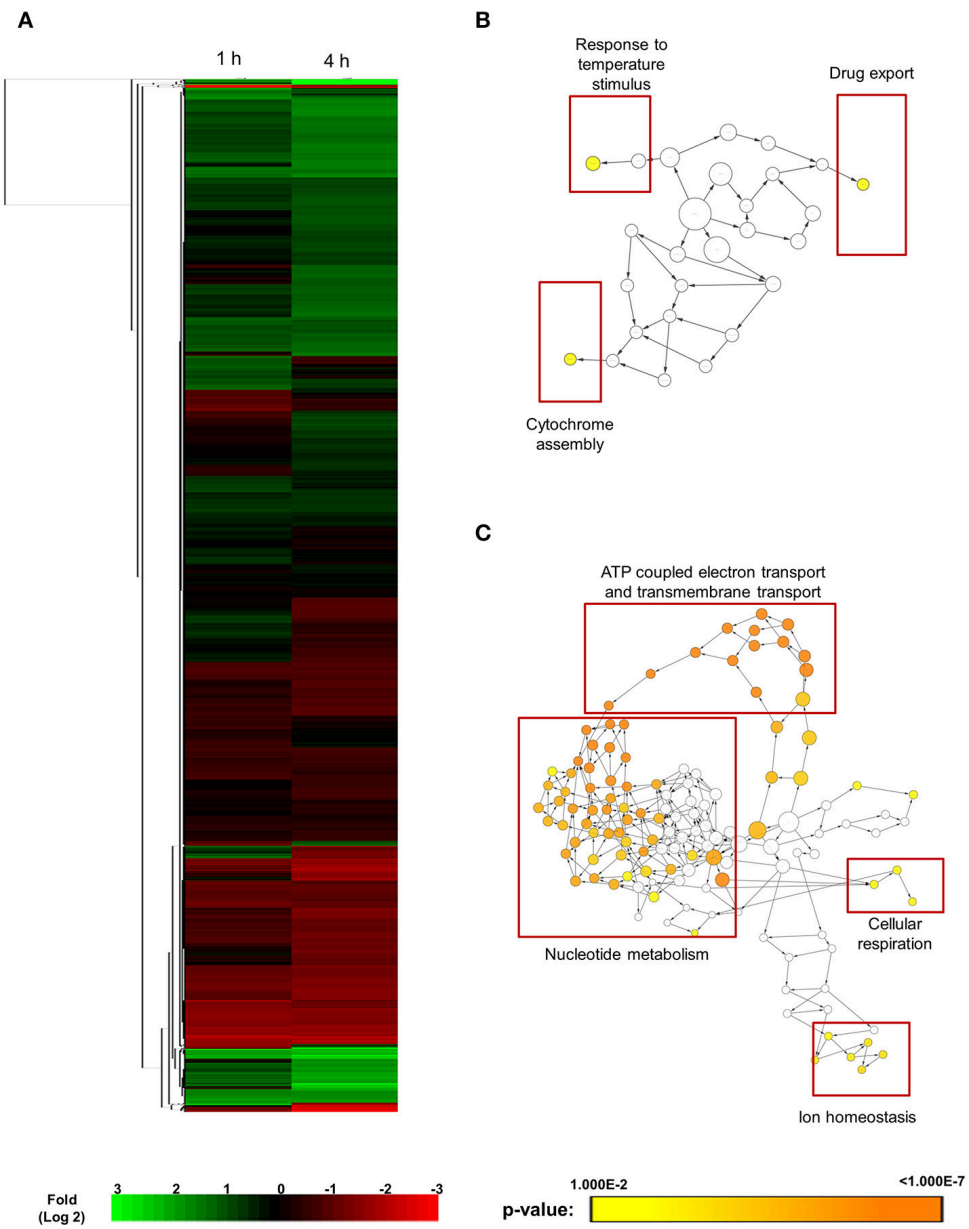
Cluster display and functional annotation of differentially expressed genes in GZY803 cultures when exposed to SM21. Overnight cultures of *C. albicans* haploid strain GZY803 at mid-log phrase (10^8^ cell/ml) were treated with SM21 (2 μg/m) for 1 or 4 h and RNA was extracted for sequencing analysis. **(A)** Clustered display of protein expression profiles of 1 or 4 h SM21-treated GZY803 strains. Biological processes, molecular functions, and cellular components that are significantly and consistently higher **(B)** or lower **(C)** in SM21-treated as compared to the untreated GZY803 strain.

SM21 was shown to be an inhibitor of the yeast-to-hypha transition of *C. albicans*, having no obvious effect on bacteria. SM21 also exhibited low toxicity to human cells (Wong et al., 2014). Hence, it is likely that the mechanism of action of SM21 is through fungal-specific targets or biological pathways. In order to understand the early lethal changes in the fungal molecular pathways induced by SM21, the *C. albicans* haploid parent strain (GZY803) was treated with 2 μg/ml of SM21, equivalent to 2 × MIC90, and RNA samples were harvested before the exposure and at 1 and 4 h. RNA was extracted from these test cultures as well as controls without SM21 treatment. Transcriptomics analysis was performed by RNA-Seq (Wang et al., 2009). Following differential expression analysis, 91 genes were classified as “higher” and 85 as “lower” in GZY803 strain under SM21 exposure compared to the untreated control. Figure 1A illustrates the expression pattern of all detected genes in a two-dimensional hierarchical clustering. Each gene was colored according to its log fold change. Next, we mapped the differently regulated proteins to their gene ontology. The analysis revealed that the expression of genes involved in response to temperature stimuli, drug export, and cytochrome assembly was significantly higher (Figure 1B) while the expression of those associated with ATP-coupled electron transport and transmembrane transport, nucleotide metabolism, cellular respiration, and ion homeostasis was significantly lower (Figure 1C) in SM21-treated GZY803 cultures compared to the untreated controls. A full list of differently expressed RNA is summarized in Table S1.

### Effects of SM21 on the mitochondria of *C. albicans* haploids

**Figure 2:**
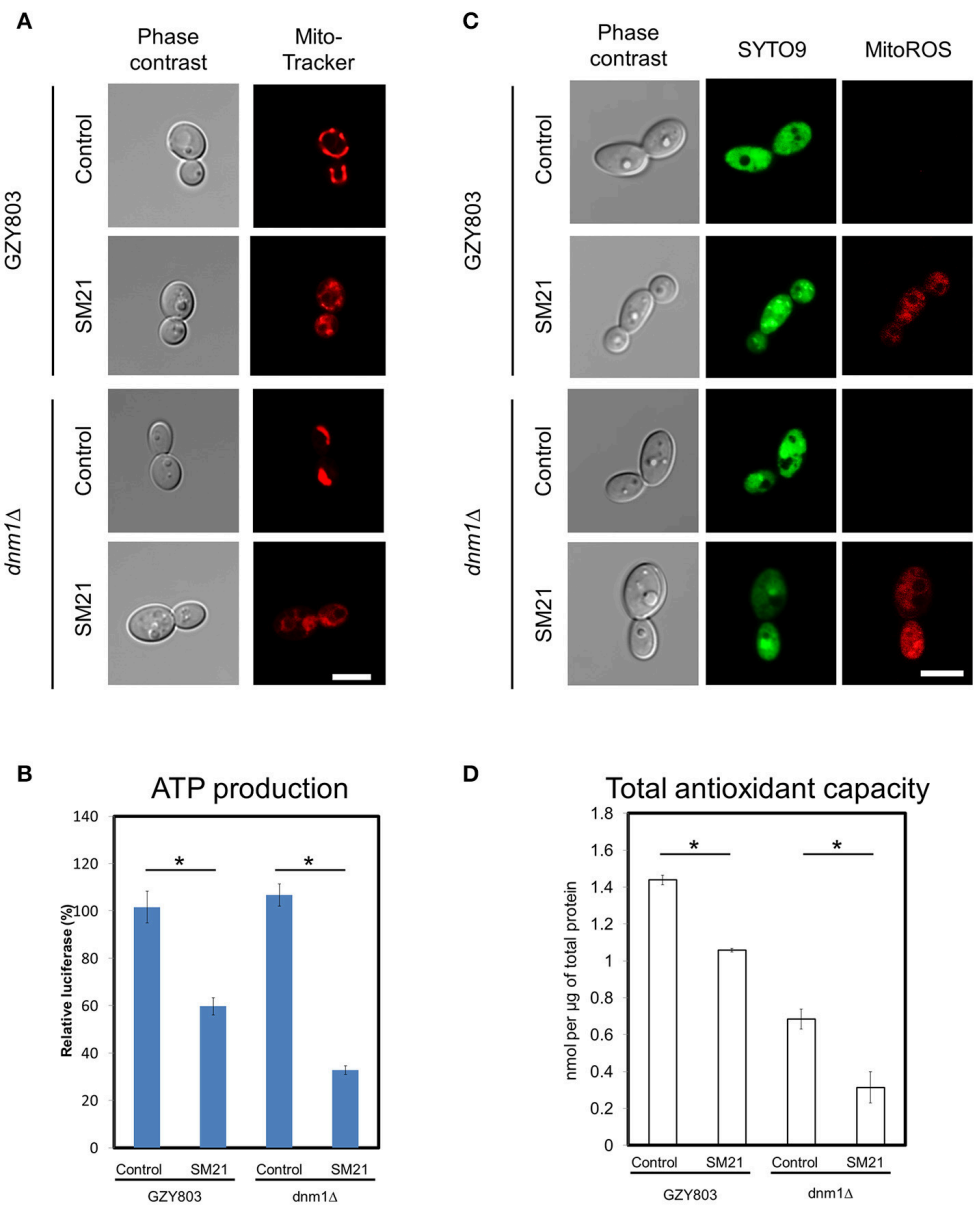
Examination of mitochondrial structures and defects in SM21-treated haploid cultures. *C. albicans* haploid GZY803 and *dnm1*Δ strains were used in the experiments. Mid-log phrase cultures (10^7^ cell/ml) were left untreated or treated with 2 μg/ml of SM21 for 4 h and cells were collected for subsequent confocal microscope visualization and ATP or antioxidant potential measurement. MitoTracker **(A)** was used to visualize the mitochondrial membrane structure under no treatment or with SM21 treatment. ATP production measurement **(B)**, ROS generation visualization via mitoSOX staining **(C)** and total antioxidant potential estimation **(D)** of treated and untreated GZY803 and *dnm1*Δ strains were also performed. Scale bar, 5 μm. For all graphs, the mean of at least three replicates is shown, with error bars showing SD. ^*^p < 0.05.

Transcriptomics studies above revealed that SM21 significantly alters the expression of genes associated with mitochondrial respiration, ATP synthesis, cytochrome assembly in haploid *C. albicans* (Figure 1, Table 1). The data suggest significant dysfunction of mitochondrial components when treated with SM21. Therefore, further downstream experiments were conducted focusing on this aspect. Firstly, mitochondrial structural changes in haploid *C. albicans* GZY803 and *dnm1*Δ strains when exposed to SM21 were examined. Dnm1 is reported to regulate mitochondrial structures in *S. cerevisiae*, and deletion of *DMN1* results in collapsed mitochondrial network with fewer branches compared to the wild type (Bleazard et al., 1999). Hence, *dnm1*Δ was included as the positive control with defective mitochondrial structures. First, *dnm1*Δ was found to be more sensitive to SM21 with a MIC90 of 0.5 μg/ml as compared to the parent GZY803 that had a MIC90 of 1 μg/ml (Table 2). Furthermore, confocal visualization of the cultures stained with MitoTracker, a dye for mitochondria, indicated that *DMN1* deletion results in compromised mitochondria with fewer branches than the wild-type GZY803. Under SM21 treatment, both GZY803 and *dnm1*Δ exhibited collapsed mitochondrial membrane potentials providing more evidence that SM21 targets mitochondria of *C. albicans* (Figure 2A). Consistently, ATP production was significantly reduced in both strains upon SM21 exposure (*p* < 0.05) with GZY803 showed a 1.5-fold reduction, and *dnm1*Δ, 3-fold, under SM21 exposure (Figure 2B). Reactive oxygen species (ROS) are the byproducts of cellular metabolism that are mostly generated at the mitochondrial level (Murphy, 2009). To examine the effect of SM21 on ROS generation and release, we stained GZY803 and *dnm1*Δ cells with MitoSOX, a florescent probe for ROS. Indeed, under SM21 treatment, ROS were released in both *dnm1*Δ and GZY803 (Figure 2C). Total antioxidant capacity measurements corroborated the observations, in which antioxidant potentials were significantly lower in *dnm1*Δ and GZY803 under SM21 treatment (*p* < 0.05) (Figure 2D). The data clearly indicate that fungal mitochondria are likely to be the target of the fungicidal effect exerted by SM21 on haploid *C. albicans*.

**Table 1:** Changes in gene expression related to complexes in electron transport chain and assembly factors in GZY803 under SM21 exposure.

**Gene**	**Function**	**Log2 fold change**	
		**1 h**	**4 h**
**NADH DEHYDROGENASE**			
NDH51	Potential mitochondrial Complex I	−1.2	−1.1
FESUR1	Potential mitochondrial Complex I	−0.8	−0.6
NDE1	Potential NADH dehydrogenase	−0.7	−0.4
YMX6	Potential NADH dehydrogenase	0.4	0.006
**COMPLEX II**			
SDH1	Succinate dehydrogenase, flavoprotein subunit	0.2	0.3
SDH2	Succinate dehydrogenase, iron-sulfur subunit	−0.4	−0.6
SDH4	Succinate dehydrogenase	0.4	0.4
**COMPLEX III**			
QCR2	Cytochrome b-c1 complex subunit 2	−1.4	−1.5
QCR7	Cytochrome b-c1 complex subunit 7	−1.2	−1.4
RIP1	Cytochrome b-c1 complex subunit Rieske	−1.2	−1.5
**COMPLEX IV**			
COX4	Cytochrome c oxidase subunit 4	−1.2	−1.8
COX7	Cytochrome c oxidase subunit 7	−0.8	−1.2
COX13	Cytochrome c oxidase subunit 6A	−1.5	−1.6
**COMPLEX V**			
ATP1	ATP synthase subunit alpha	−1.3	−1.7
ATP2	ATP synthase subunit beta	−1.1	−1.5
ATP3	ATP synthase subunit gamma	−1.3	−1.6
ATP4	F1F0 ATP synthase subunit 4	−1	−1.5
ATP5	F1F0 ATP synthase subunit 5	−1.2	−1.4
ATP7	ATP synthase subunit d	−1.3	−1.7
ATP16	F1F0 ATP synthase subunit delta	−0.4	−0.6
ATP20	F1F0 ATP synthase subunit g	−1.3	−1.6
**ASSEMBLY FACTORS**			
orf19.4324	Possible mitochondrial Complex I	0.6	0.3
orf19.4727	Succinate dehydrogenase assembly factor 2	0.4	0.1
SHD7	Succinate dehydrogenase assembly factor 3	0	−0.1
CBP4	Assembly factor CBP4	0.9	1.1
COX11	Uncharacterized protein Cox11p	0.8	0.6
COX15	Uncharacterized protein Cox15p	1.4	1.4
COX16	Cytochrome c oxidase assembly protein COX16	0.5	0.6
COX19	Cytochrome c oxidase assembly protein COX19	0.9	1
COX20	Cytochrome c oxidase protein 20	0.2	0.2
COX23	Cytochrome c oxidase-assembly factor COX23	0.9	0.7
ATP12	ATP synthase complex assembly protein	−0.00006	−0.008

**Table 2:** Minimum inhibition concentration of *Candida albicans* strains.

**Minimum inhibiting concentration (MIC90)**						
**Antifungal**	**SC5314**	**GZY803**	***dnm1**Δ*	**S3**	**S5**	**S6**
SM21	1	1	0.5	4	4	4
Amphotericin B	0.25	0.25	0.25	0.25	0.25	0.25
Caspofungin	0.5	0.5	NT	0.5	0.5	0.5
Fluconazole	0.5	0.25	NT	0.25	0.25	0.25
Ketoconazole	0.125	0.125	NT	0.125	0.125	0.125
Voriconazole	0.006	0.006	NT	0.006	0.006	0.006

*NT, Not tested*.

### Confirmation of SM21 inhibitory effects on mitochondria of *C. albicans* diploid cells

**Figure 3:**
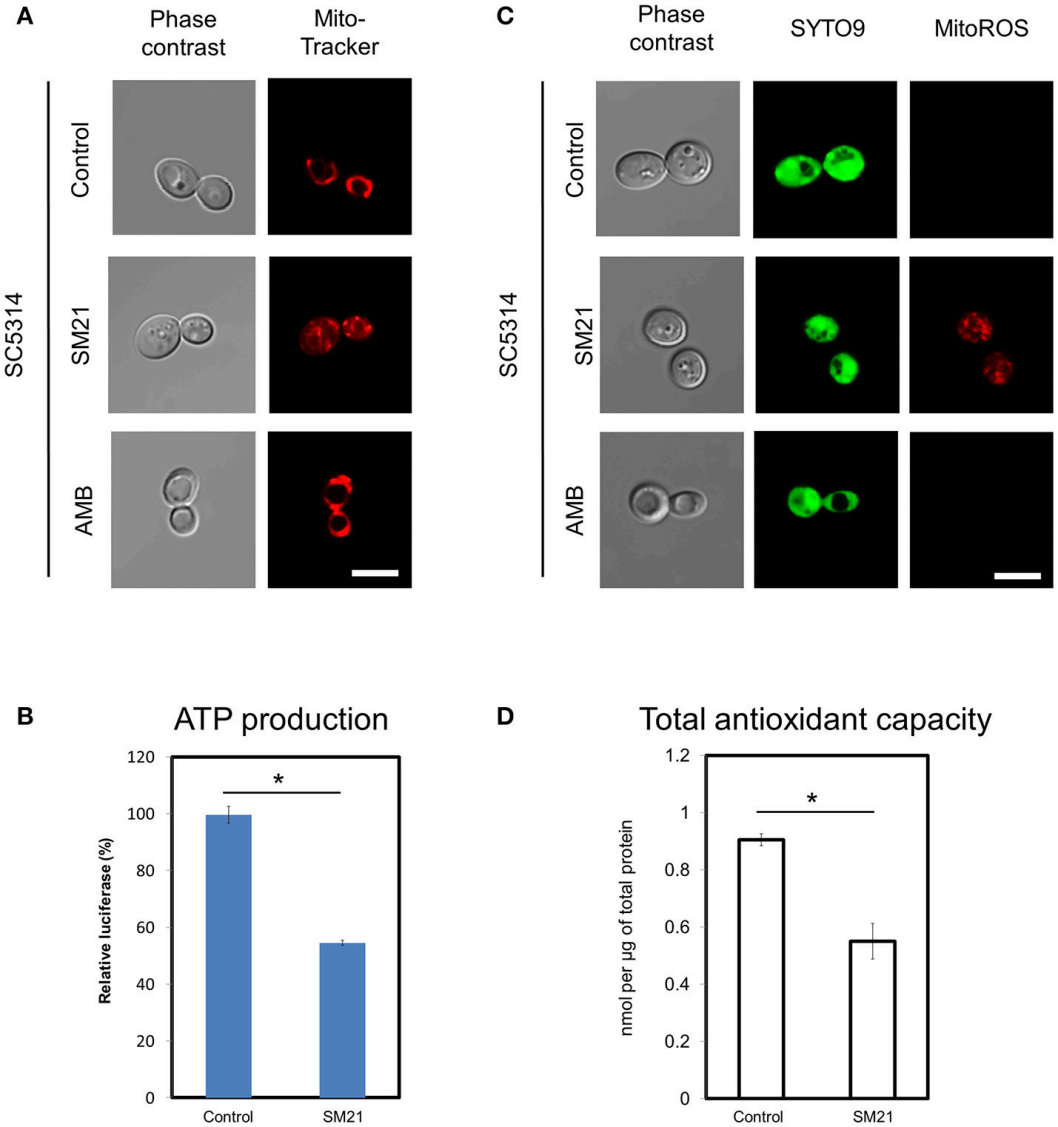
Changes in mitochondrial structures and functions of *C. albicans* diploids under SM21 exposure. *C. albicans* diploid strain, SC5314, at mid-log phrase (10^7^ cell/ml) was used in the experiments. Cultures were left untreated or treated with 2 μg/ml of SM21 for 4 h and cells were collected for subsequent visualization of mitochondrial structure **(A)**, ATP measurement **(B)**, ROS release visualization **(C)**, and antioxidant potential examination **(D)**. Treatment of SC5314 cultures with amphotericin B (2 μg/ml, 4 h) was also included in confocal microscope visualization to serve as the control. Scale bar, 5 μm. For all graphs, the mean of at least three replicates is shown, with error bars showing SD. ^*^p < 0.05.

In order to confirm mitochondrial defects in *C. albicans* diploids, we examined the changes of mitochondrial functions of a standard diploid strain, SC5314, under SM21 treatment. Consistent with the above findings, SC5314 exhibited collapsed mitochondrial membrane potentials when treated with SM21 but not with amphotericin B (Figure 3A). Under SM21 treatment, SC5314 also displayed a reduction in ATP generation (*p* < 0.05) (Figure 3B), an increase in ROS generation (Figure 3C), and a corresponding decrease in antioxidant potential (*p* < 0.05) (Figure 3D). These data, therefore, confirmed that SM21 inhibits mitochondria activity in *C. albicans*.

### Generation of SM21-resistant haploid strains

**Figure 4:**
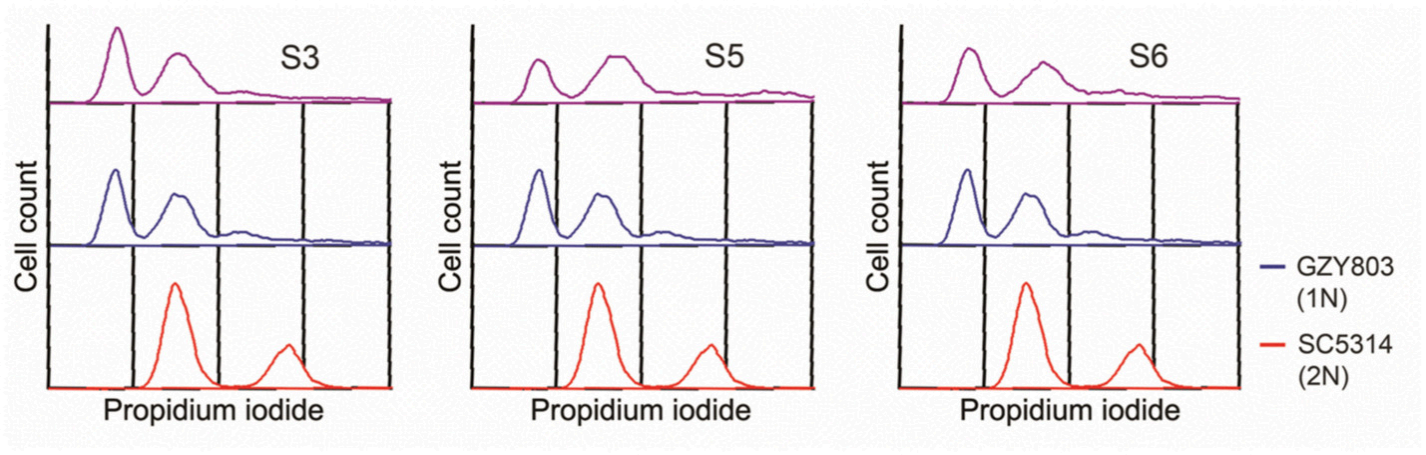
Ploidy examination of resistant haploid strains of *C. albicans*. SM21-resistant haploid cells were grown at 30°C overnight. Subsequently, mid-log phrase cultures were taken for ploidy examination by flow cytometry analysis. SC5314 and GZY803 were used as the standard for diploid and haploid genomes.

In order to understand the drug-pathogen interaction, it is important to know the mechanisms of action of the antimicrobial as well as the resistance mechanisms expressed by the pathogen against it. Therefore, as the next step, taking advantage of the haploid *C. albicans* model, we generated SM21-resistant mutants from the standard haploid parent GZY803. An inoculum of 10^8^ cells of GZY803 were grown in GMM broth supplemented with 2 μg/ml of SM21 for 3 consecutive days with the SM21-containing medium refreshed every 24 h; surviving cells were then spread on Sabouraud dextrose agar plates containing 4 μg/ml of SM21. Among the colonies developed, three colonies designated as S3, S5, and S6, displayed a SM21 MIC of 4 μg/ml and were selected for further studies. Other colonies having a MIC < 4 μg/ml were discarded. Interestingly, all three colonies resistant to SM21 exhibited similar susceptibility to other antifungals tested as the wild-type strain (Table 2). This suggested that the resistance mechanisms possessed by the resistant haploid *C. albicans* colonies are effective against SM21 but not to the other antifungals tested. Susceptibility of the resistant strains to SM21 as well as other antifungals were repeatedly examined in several different occasions and in parallel with any other experiments performed to ensure that resistances acquired in the strains are non-reversal. In addition, FACS ploidy examination was also performed which revealed that all of the resistant strains maintained the haploidy (Figure 4). Therefore, using the haploid model of *C. albicans* is a suitable strategy to uncover resistance mechanisms.

### Transcriptomic analysis on SM21-resistant haploid strains

**Figure 5:**
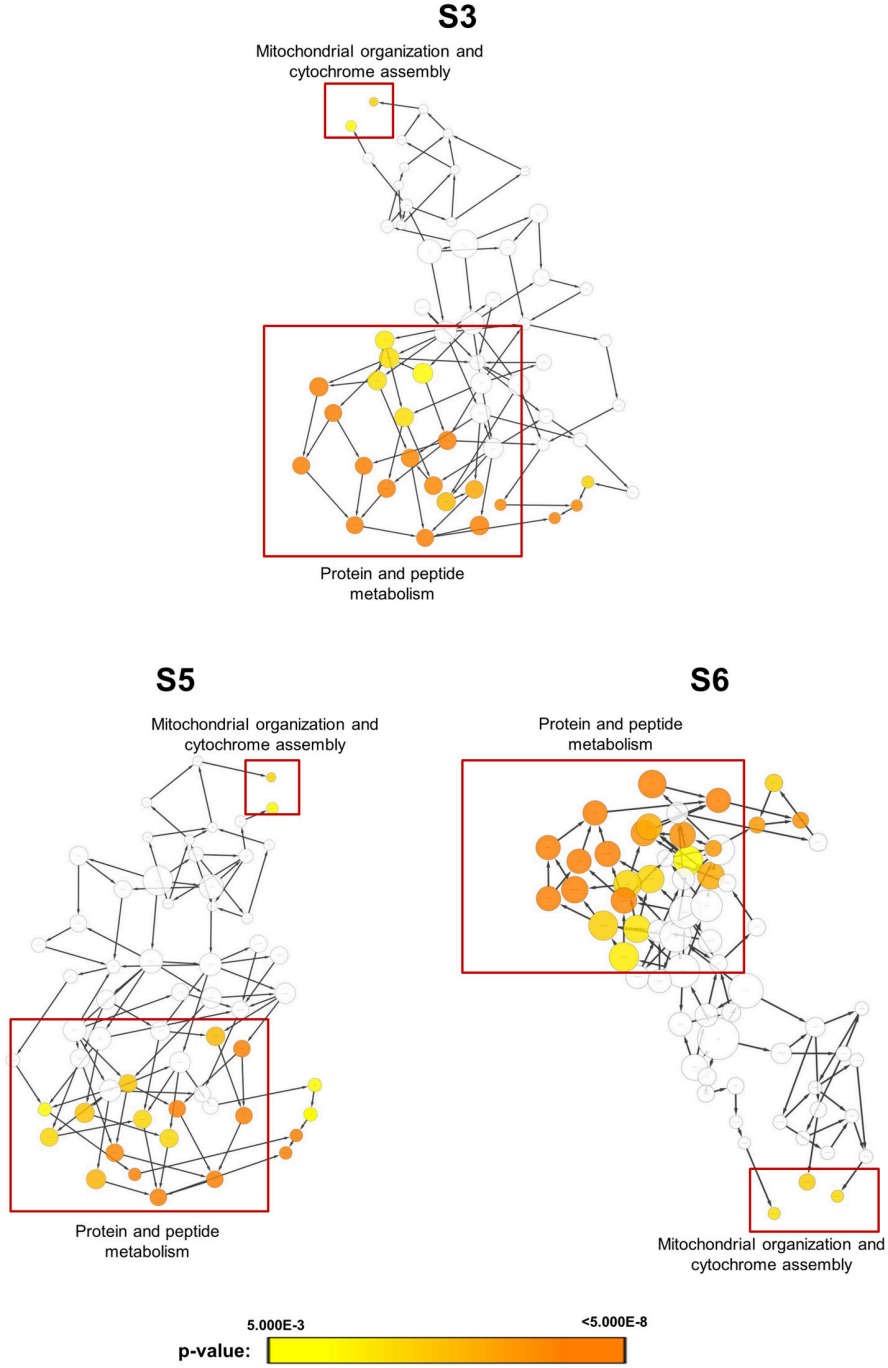
Gene ontology analysis of genes with higher expression in resistant strains when exposed to SM21. SM21-resistant haploid strains at mid-log phrase (10^8^ cell/ml) were treated with 2 μg/ml of SM21 for 1 or 4 h. RNA was extracted and sent for RNA sequencing. Differential expression analysis was first applied to determined genes which were upregulated in the resistant haploid strains under SM21 exposure. Subsequent gene ontology analysis illustrated biological processes which were significantly higher in the resistant haploid strains under SM21 treatment.

**Figure 6:**
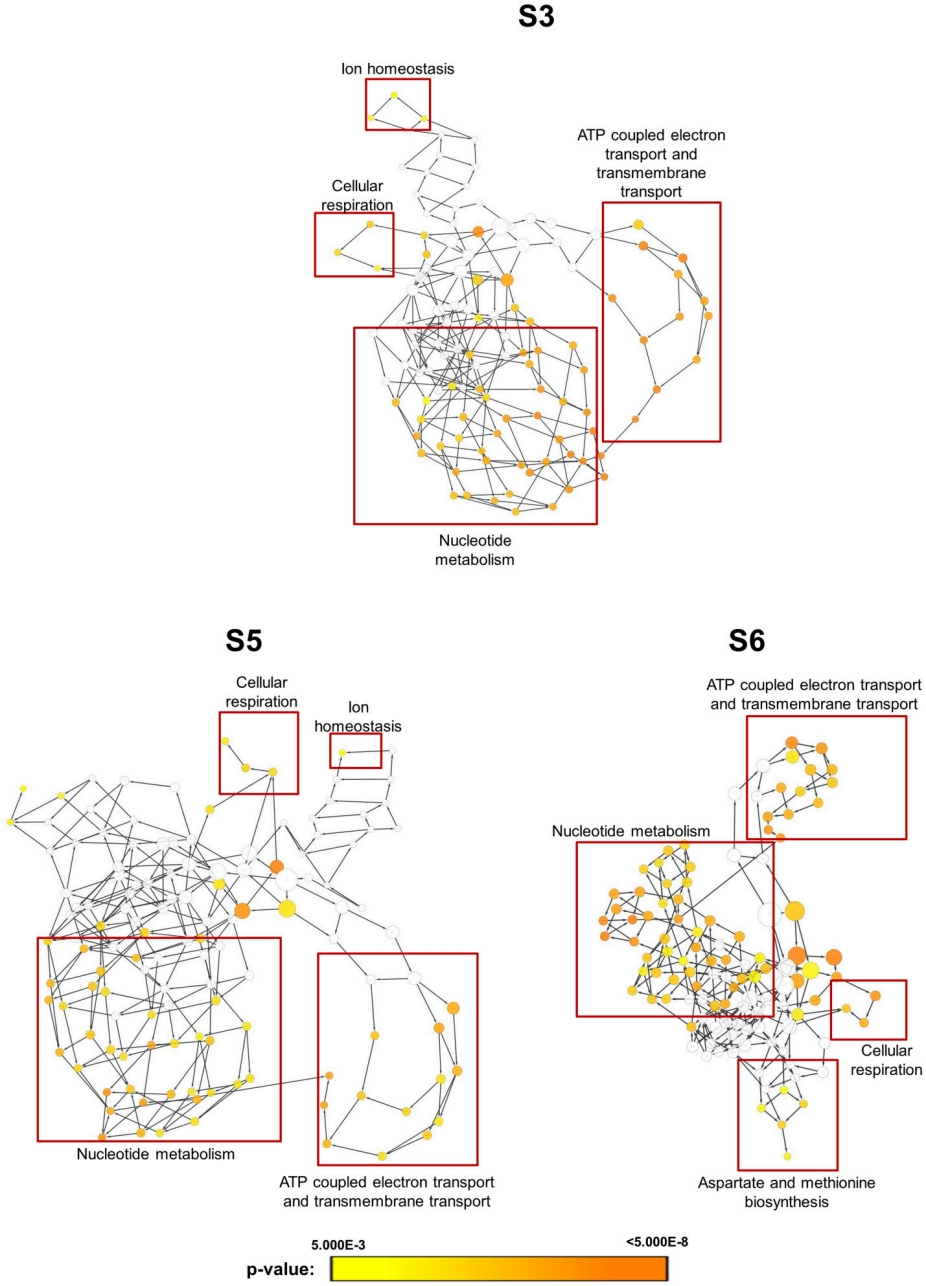
Gene ontology analysis of genes with lower expression in resistant strains when exposed to SM21. SM21-resistant haploid strains at mid-log phrase (10^8^ cell/ml) were treated with 2 μg/ml of SM21 for 1 or 4 h. RNA was extracted and sent for RNA sequencing. Differential expression analysis was first applied to determine genes which were downregulated in the resistant haploid strains under SM21 exposure. Subsequent gene ontology analysis illustrated biological processes which were significantly lower in the resistant haploid strains under SM21 stimulant.

**Figure 7:**
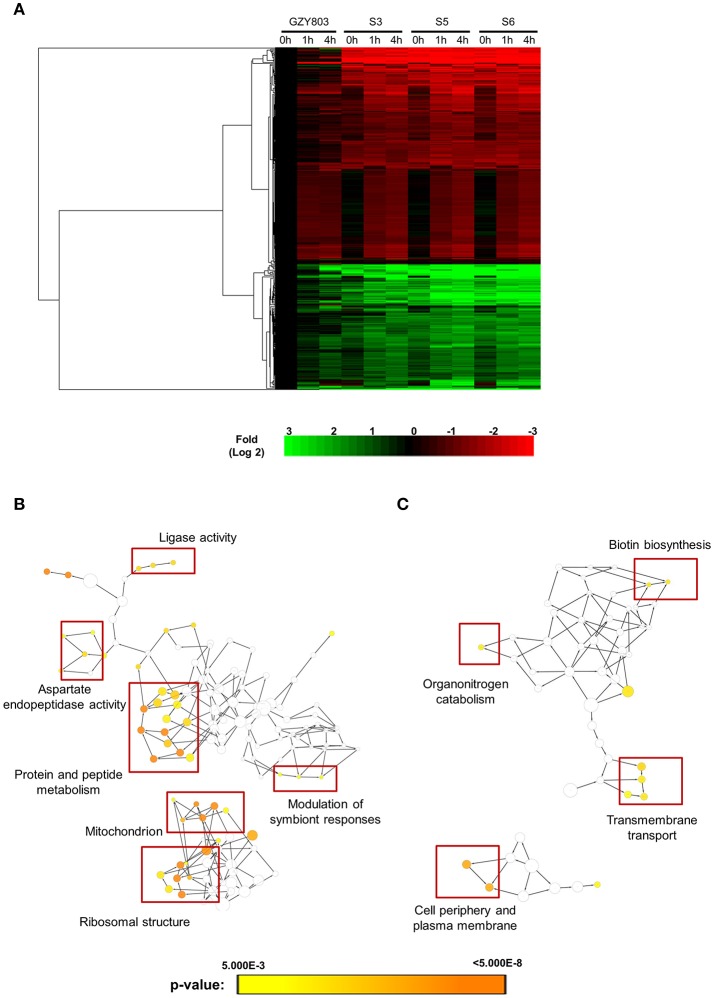
Cluster display and functional analysis of gene expression changes in resistant strains relative to that of GZY803. SM21-resistant haploid strains and GZY803 at mid-log phrase (10^8^ cell/ml) were treated with 2 μg/ml of SM21 for 1 or 4 h. Subsequently, RNA were extracted and sent for sequencing. RNA from untreated cultures was also collected as a control. **(A)** Cluster display of genes exhibited significant changes in fold expression in the resistant strains as compared to GZY803. Biological processes which are significantly upregulated **(B)** or downregulated **(C)** in the resistant strains compared to the wild-type control are shown.

In order to obtain clues on the resistance mechanism, the resistant haploid strains were treated with SM21 together with untreated controls to obtain RNA for RNA-Seq analysis. Subsequent transcriptomic examination involved (1) analysis of genes differently expressed in each resistant haploid strain under control and SM21 treatment; and (2) determination of genes which showed a similar expression trends in all resistant strains but were differently expressed compared to that in GZY803 under the same conditions. First step of differential analyses found that genes related to mitochondrial organization, cytochrome assembly, and protein and peptide metabolism were upregulated (Figure 5), while genes involved in cellular respiration, and ATP-coupled electron and transmembrane transports, downregulated, in all resistant strains under SM21 treatment (Figure 6). This suggested that all the resistant strains were affected by SM21 inhibitory effects on mitochondrial activity. Among the strains, under SM21 treatment, S3 and S5 displayed lower expression of genes involved in ion homeostasis, and S6, aspartate and methionine biosynthesis (Figure 6). Next, we applied Limma differential gene expression analysis to identify genes commonly expressed in all the resistant strains, which, on the other hand, were differently expressed in GZY803 under the same conditions (Ritchie et al., 2015). The method identified 211 genes whose expressions were higher, and 122 genes, lower, in the resistant haploid strains as compared to GZY803 (Table S2). Figure 7A illustrated clustering of differently expressed genes in the SM21 resistant haploid strains as compared to GZY803. Functional annotation of these genes revealed that expressions of genes modulating ligase activity, aspartate endopeptidases, protein and peptide metabolism, symbiont responses, mitochondrion, and ribosome structures were higher, and expression of genes involved in biotin biosynthesis, organonitrogen catabolism, transmembrane transport, cell periphery, and plasma membrane was lower in the resistant strains than in GZY803 in un-treated or SM21 exposed conditions (Figures 7B,C).

### Exploring the role of amino acid supplements in SM21 resistance

The first set of foregoing experiments found that SM21 inhibits the activities of mitochondria, which are also known to be involved in amino acid synthesis (Palmieri et al., 1997; Birsoy et al., 2015). The second set of experiments suggested that the resistant strains exhibited SM21-induced transcriptomic inhibition of mitochondrial activity but upregulated genes related to peptide metabolism under the treatment. In addition, the resistant cells also expressed higher level of genes encoding secreted aspartic protease activity as compared to GZY803. Secreted aspartic proteases (SAPs) are a family of at least 10 members (Naglik et al., 2003) which have been shown to degrade host tissues and proteins and peptides for nutrient amino acids supplement and invasion (Schaller et al., 2003). Taking the above findings into account, we hypothesized that there is a link between amino acid supplementation and SM21 resistance in *C. albicans*. To test the hypothesis, we supplied the wild-type and resistant *C. albicans* cells with peptone, a form of digested peptides from animal tissues, and examined the susceptibility changes of the strains. Indeed, supplementing the haploid GZY803 and the diploid SC5315 strains with peptone increased the MIC90 of *C. albicans* against SM21 from 1 to 2 μg/ml (Table 3). Adding 10% of peptone into the cultures of the resistant strains also increased their SM21 MIC90 from 4 to 8 μg/ml. On the contrary, there was no change in MIC90 when peptone supplement was provided to the amphoterin B-treated cultures of *C. albicans* (Table 3). In conclusion, our data have shown that improving amino acid supplement plays a role in SM21 resistance in *C. albicans*.

**Table 3:** Minimum inhibition concentration of *Candida albicans* strains under 10% peptone supplement.

**Minimum inhibiting concentration (MIC90)**						
**Antifungal**		**SC5314**	**GZY803**	**S3**	**S5**	**S6**
SM21	Without peptone	1	1	4	4	4
	10% peptone	2	2	8	8	8
Amphotericin B	Without peptone	0.25	0.25	0.25	0.25	0.25
	10% peptone	0.25	0.25	0.25	0.25	0.25

## Discussion

The recent isolation of *C. albicans* haploids has shown its utility in genetic and biofilm studies (Seneviratne et al., 2015; Truong et al., 2016). Herein, we provide the first demonstration of the use of haploid *C. albicans* model in elucidating the mechanism of action of a novel antifungal agent SM21. Initially, we obtained the transcriptomic profiles of haploid *C. albicans* in response to SM21. These analyses provided vital information about the possible mechanism of action of SM21, indicating fungal mitochondria as a potential target. Further downstream experiments using both haploid and diploid *C. albicans* provided substantial evidence on this claim. Moreover, use of haploid *C. albicans* helped us to efficiently generate mutations and isolate SM21-resistant strains. Hence, this proof-of-concept study has clearly demonstrated the advantages of the haploid model of *C. albicans* to the fungal research community. Interestingly, not only in *C. albicans*, uses of haploid cells have been exploited in many organisms. Due to its advantages of allowing simpler genetic manipulation, haploid cells have been used widely in recessive phenotype analysis in various species including the yeast *S. cerevisae*, bees, and some type of plants, in which haploid cells exist naturally as part of their life cycle (Haber, 2012; Duina et al., 2014). Even in mammals, where haploid state is only restricted to gametes, haploid embryos have been used for genetic screening (Elling et al., 2011; Yang et al., 2012). Hence, the new haploid model of *C. albicans* will be a useful tool for researchers working on antifungal drug discovery studies.

Using haploid *C. albicans*, we found that SM21 inhibits fungal mitochondria and alters mitochondrial activity. SM21 inhibited ATP synthesis in fungal cells via reducing the expression of genes in the ATP synthesis machinery (Figures 1–3). ATP is a necessary form of energy in all types of eukaryotic cells (Kaim and Dimroth, 1999; von Ballmoos et al., 2009). Therefore, inhibiting ATP generation in *Candida* cells would be lethal to the fungal pathogen. Limiting ATP-coupled electron transportation leads to increased electron leakage, causing accumulation of ROS inside *Candida* cells (Murphy, 2009; Divakaruni and Brand, 2011). Increased ROS levels causes damages to proteins, DNA, and lipids, thereby compromising *C. albicans* viability (Cabiscol et al., 2000). In addition, lipid peroxidation which occurs as a result of the reaction of free radicals leads to damage in cell membranes (Dantas Ada et al., 2015). In fact, this is in line with our previous observations of the activity of SM21 on *C. albicans* (Wong et al., 2014). High levels of ROS is also a signal for *Candida* cells to undergo apoptosis (Perrone et al., 2008). In addition, SM21 treatment also altered membrane potentials of *C. albicans* haploid and diploid cells (Figures 2, 3). Therefore, it is likely that SM21 also induces changes in mitochondrial morphology, which in turn is the signal to cell death (Ishihara et al., 2003; Kubli and Gustafsson, 2012; Song et al., 2017). However, further experiments using targeted mitochondrial proteins are warranted to obtain conclusive evidence.

Previously, it was shown that SM21 is fungal-specific and has no activity on bacteria (Wong et al., 2014). Findings in the present study show that SM21 inhibits fungal cytochrome and ATPases, explaining the foregoing observation (Figures 1–3, Table 1). Cytochromes are proteins embedded in the inner mitochondrial membrane of eukaryotes (Ow et al., 2008). Cytochromes transport protons from the mitochondrial matrix into the intermembrane space, generating an electron gradient for ATPases to synthesize ATP (Ow et al., 2008). SM21, by inhibiting cytochrome gene expression, causes reduction in proton transport and ATP synthesis in the fungal cells. Bacteria, on the other hand, do not possess any membrane-bound organelle like mitochondria, instead, rely on a different machinery to generate energy. Besides glycolysis, bacteria utilize various flavins or non-heme iron components for generating gradients across their cytoplasmic membrane for ATP synthesis (Jurtshuk, 1996). This explains why SM21 is not effective for bacteria.

Targeting fungal mitochondria was recently proposed to be a promising therapeutic approach for developing novel antifungal agents. A study by Dagley et al. (2011) demonstrated the involvement of *C. albicans* Sam37, a mitochondrial outer-membrane protein, in caspofungin susceptibility (Dagley et al., 2011). Similarly, mitochondrial mutants in *C. glabrata* were found to be associated with increased susceptibility to amphotericin B (Vandeputte et al., 2009). Likewise, inactivation of the mitochondrial protein Goa1, which is required for mitochondrial biogenesis, leads to reduced virulence in systemic candidiasis mouse model (Bambach et al., 2009; Becker et al., 2010). Furthermore, several fungal mitochondrial factors do not have close orthologs in humans (Shingu-Vazquez and Traven, 2011). Therefore, targeting mitochondrial functions could be an effective novel antifungal therapy against *Candida*, especially for the isolates that display resistance to existing antifungal agents. More evidence on this aspect has come from recent studies of natural compounds which inhibit the activity of *C. albicans* mitochondria, but not toxic to human cells (Lee et al., 2017; Tian et al., 2017). Similarly SM21 was also shown to be non-toxic to human cell lines (Wong et al., 2014), although it causes mitochondrial dysfunction in fungal cells. In addition to that, SM21 displays fungicidal properties against multidrug resistant *Candida* strains, such as the T 1549 *C. guilliermondii* isolates (Wong et al., 2014). Hence, SM21 is proven to be a substantial and effective antifungal molecule for future clinical anti-*Candida* drug development.

Further in this study, using the *C. albicans* haploid strains, we have developed SM21 haploid resistant strains and uncovered different pathways altered in the resistant cultures under SM21 treatment relative to GZY803 (Figures 5–7). From the changes observed, we examined and validated the involvement of amino acid supplements in SM21 resistance (Table 3). Mitochondria are known to be the site of many important metabolic and biosynthetic reactions, such as the tricarboxylic acid (TCA) cycle and synthesis of certain essential amino acids (Epstein et al., 2001). Indeed, failure of amino acid homeostasis results in cell death following proteasome inhibition in yeast (Suraweera et al., 2012). Hence, it is possible that amino acid supplements, by compensating for the loss at mitochondrial level, increases the survival rate under SM21 treatment. In addition, all resistant strains have been found to display higher gene expression for SAP activity than GZY803 (Figure 7). Increased SAP activity has been reported in *C. albicans* when exposed to antifungal agents such as fluconazole, itriconazole, and caspofungin (Copping et al., 2005). SAPs are an important virulence attribute of *C. albicans* and SAP-deletion strains have been shown to be less virulent and lack tissue invasion (Sanglard et al., 1997; Schaller et al., 2003). SAP is also crucial in providing nitrogen nutrient to *Candida* cells (Brown et al., 2014; Ramachandra et al., 2014). The information is in line with our hypothesis that higher levels of amino acid supplements benefit the resistance to SM21 in *C. albicans*. SAP expression is known to be induced by Efg1 and Cph1 and suppressed by Tup1 transcriptional factors (Naglik et al., 2004). The mechanism behind SAP regulation in SM21 resistance in *C. albicans* is an interesting aspect yet to be uncovered.

In conclusion, this study has shown, for the first time, the use of haploid *C. albicans* to uncover the mechanism of action of novel antifungal SM21 and *C. albicans*' resistance mechanism against SM21. Identification of SM21 as an inhibitor of fungal mitochondrial activity provides critical information to the development of this small molecule as a new antifungal agent in future clinical studies. Further experiments using targeted fungal mitochondrial proteins are warranted to obtain conclusive evidence on the proposed mechanism of action. The new insight given by this study on the new haploid *C. albicans* model will be invaluable for researchers working on antifungal drug discovery.

## Availability of data and materials

The data discussed in this publication have been deposited in NCBI's Gene Expression Omnibus (Edgar et al., 2002) and are accessible through GEO Series accession number GSE104900 (https://www.ncbi.nlm.nih.gov/geo/query/acc.cgi?acc=GSE104900).

## Author contributions

CS, YW, and TT conceived, designed the experiments, and co-wrote the manuscript. TT performed the drug treatment, resistant mutation generation, RNA extraction, bioinformatics analysis, confocal imaging, and other biochemical experiments. GZ generated some fungal strains and performed the FACS ploidy examination. TS helped in the DNA extraction. JL, TL, and LL gave guidance and assisted in the bioinformatics analysis. CT gave general advices and assisted in the experiments. All authors revised the manuscript.

### Conflict of interest statement

The authors declare that the research was conducted in the absence of any commercial or financial relationships that could be construed as a potential conflict of interest. The reviewer AC and handling Editor declared their shared affiliation.
